# Construction and model-based analysis of a promoter library for *E. coli*: an indispensable tool for metabolic engineering

**DOI:** 10.1186/1472-6750-7-34

**Published:** 2007-06-18

**Authors:** Marjan De Mey, Jo Maertens, Gaspard J Lequeux, Wim K Soetaert, Erick J Vandamme

**Affiliations:** 1 Department of Biochemical and Microbial Technology, Faculty of Bioscience Engineering, Ghent University, Coupure Links 653, 9000 Ghent, Belgium; 2 BIOMATH, Department of Applied Mathematics, Biometrics and Process Control, Faculty of Bioscience Engineering, Ghent University, Coupure Links 653, 9000 Ghent, Belgium

## Abstract

**Background:**

Nowadays, the focus in metabolic engineering research is shifting from massive overexpression and inactivation of genes towards the model-based fine tuning of gene expression. In this context, the construction of a library of synthetic promoters of *Escherichia coli *as a useful tool for fine tuning gene expression is discussed here.

**Results:**

A degenerated oligonucleotide sequence that encodes consensus sequences for *E. coli *promoters separated by spacers of random sequences has been designed and synthesized. This 57 bp long sequence contains 24 conserved, 13 semi-conserved (W, R and D) and 20 random nucleotides. This mixture of DNA fragments was cloned into a promoter probing vector (pVIK165). The ligation mixtures were transformed into competent *E. coli *MA8 and the resulting clones were screened for GFP activity by measuring the relative fluorescence units; some clones produced high fluorescence intensity, others weak fluorescence intensity. The clones cover a range of promoter activities from 21.79 RFU/OD_600 _ml to 7606.83 RFU/OD_600 _ml. 57 promoters were sequenced and used for promoter analysis. The present results conclusively show that the postulates, which link promoter strength to anomalies in the -10 box and/or -35 box, and to the length of the spacer, are not generally valid. However, by applying Partial Least Squares regression, a model describing the promoter strength was built and validated.

**Conclusion:**

For *Escherichia coli*, the promoter strength can not been linked to anomalies in the -10 box and/or -35 box, and to the length of the spacer. Also a probabilistic approach to relate the promoter sequence to its strength has some drawbacks. However, by applying Partial Least Squares regression, a good correlation was found between promoter sequence and promoter strength. This PLS model can be a useful tool to rationally design a suitable promoter in order to fine tune gene expression.

## Background

Metabolic engineering is hardly a decade old but its significance is already generally recognized. Metabolic engineering is nowadays commonly applied to improve the properties and performances of industrial microorganisms: to improve general cellular properties, to increase the yield and the productivity of native microbial products and for the synthesis of products that are new to the host cell [1,2].

Thus far, metabolic engineering has been largely restricted to the deletion and/or massive overexpression of genes involved in byproduct formation or in the rate determining steps of a metabolic pathway. However, in some cases such drastic modifications result in deteriorated strain performances, as the resulting flux distribution of such an intervention might not be optimal anymore, due to the interplay of the metabolic pathways in the producer strain.

Therefore, more rigorous techniques are used both experimentally [3,4] and mathematically [5-7] to both identify and remedy the bottlenecks in a metabolic pathway. In addition metabolic control analysis has pointed out that the control and regulation of cellular metabolism is distributed over several enzymes in a pathway [8]. Multiple modifications in order to alter the expression level of the enzymes might thus be mandatory in order to obtain the desired yield increase.

These mathematical techniques comprise amongst others the use of detailed dynamic models, both mechanistic and approximate ones, which are able to elucidate the rate determining steps in a metabolic pathway. With respect to the experimental techniques, the construction of promoter libraries seems promising [5,9-16].

Several inducible expression systems are now available for *Escherichia coli*. These systems need addition of an inducer to have promoter activity. In the presence of an inducer, expression should vary directly and preferably linearly with the level of added inducer. Unfortunately, most expression systems seem to exhibit an all-or-nothing phenomenon. Though the population-averaged expression of a gene controlled by an inducible promoter varies roughly linearly with the amount of inducer, it is found to be fully induced in a fraction of the cells and not induced in the remaining cells [17]. However for metabolic engineering purposes all cells in a culture should be induced uniformly. Such inducers are thus not fit for fine tuning gene expression in order to redirect the flux towards the desired product.

An alternative to the inducible expression systems would be to insert a constitutive promoter that has the exact optimal strength. However there is a lack of constitutive promoters for *E. coli *and the available ones do not differ much in strength. In the literature [9,13,15,16], different methods are described for generating libraries of artificial promoters for a selected microorganism or group of organisms. The promoter libraries cover a wide range of promoter activities, in small steps of activity increase. We have followed the strategy of Jensen & Hammer (1998b) [13] to construct a library of synthetic promoters as a useful tool for fine tuning gene expression in *Escherichia coli*.

Finally, different mathematical techniques were applied to find a correlation between promoter strength and promoter sequence.

## Results and discussion

The purpose of this work was to construct a library of artificial constitutive promoters as a useful tool for the model-based fine tuning of gene expression in *Escherichia coli*. The synthetic promoters should cover a wide range of promoter activities. According to the procedures of Jensen & Hammer (1998a, 1998b) [12,13], the following strategy was performed: (1) design and synthesize a degenerated oligonucleotide sequence that encodes consensus sequences for *E. coli *promoters, separated by spacers of random sequences and flanked with non-degenerated multi cloning sites; (2) convert this mixture of degenerated oligonucleotides to double-stranded DNA-fragments using the Klenow fragment of DNA polymerase I and a short oligonucleotide primer complementary to the 3' of the non-degenerated flank; and (3) clone this mixture of degenerated DNA fragments into a promoter probing vector.

### Design and construction of a degenerated oligonucleotide sequence

**Figure 1:**
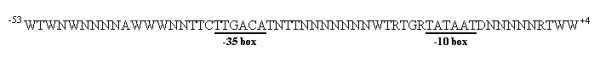
Consensus sequence for *E. coli *promoters derived after literature review. D = 33.33% each A, C and G; N = 25% each A, C, G and T; R = 50% each A and G; W = 50% each A and T.

The sequence of promoters of *E. coli *has been described and analyzed in several reports [18-25]. Also consensus sequence motifs are known for prokaryotic promoters [12,13,26]. From these data we extracted the consensus sequence for an *E. coli *promoter (see figure 1).

The Pribnow box or -10 box TATAAT and the -35 box TTGACA are well known to be present in many prokaryotic promoters and are well conserved for *E. coli*. In addition, the sequence TTC and TNTT are often found immediately upstream and downstream the -35 box, respectively. The sequence TG is frequently found 1 bp immediately upstream the -10 box. Further the base pair A and T are found at position -44 and -52, -17 and +2, respectively. In addition to these motifs 3 semi-conserved base pairs were included, D (= A, C or G) downstream the -10 box, R (= A or G) at position -16, -13 and +1 and W (= A or T) at position -53, -51, -49, -43, -42, -41, -18, +3 and +4. Based on these data a 57 bp long sequence containing 24 conserved (A, G, C and T), 13 semi-conserved (W, R and D) and 20 random nucleotides (N) was designed.

**Figure 2:**
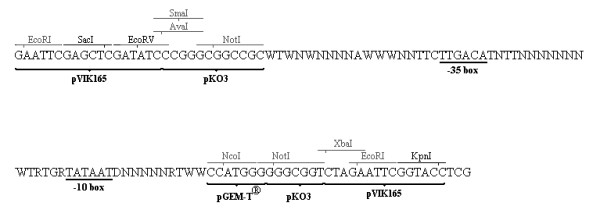
The degenerated oligonucleotide sequence designed for the construction of an *E. coli *promoter bank. The sequence contains a number of recognition sequences for restriction endonucleases, for use in the subsequent cloning strategy.

In order to obtain an artificial promoter library suitable for cloning, non-degenerated flanks that carry multiple recognition sites for restriction endonucleases (Multiple Cloning Site MCS) were added to both ends of the single-stranded DNA sequence. This resulted in an oligonucleotide sequence of 119 nt (see figure 2). This sequence was synthesized by Sigma Genosys^®^. The mixture of oligonucleotides was then converted to double-stranded DNA-fragments, using Klenow fragment of DNA polymerase and a short oligonucleotide primer complementary to the 3' end of the non-degenerated flank. In the next step, this mixture of DNA fragments encoding potential promoter structures was cloned into a promoter probing vector (pVIK165) using the following cloning strategy: the mixture of degenerated promoter oligonucleotides and pVIK165 were digested with restriction enzymes SacI and XbaI and the degenerated promoter fragments were ligated to the compatible vector fragments. The ligation mixtures were transformed into competent *E. coli *MA8 cells resulting in several clones.

### Development of a green fluorescent protein assay

An assay for green fluorescent protein on liquid cultures was developed based on the GFP assay described by Clontech Laboratories (1999) and Gonzales (2005) [27,28]. First, the relative fluorescence of LB-cultures was compared to LB-cultures where the cells were disrupted by sonication or 1 drop of 0.1% SDS (sodium dodecyl sulphate). However, there was no difference in fluorescence between the different procedures. Because LB shows auto fluorescence, the cells were harvested and washed and solved in TE-buffer (10 mM Tris-HCl, pH 7.5, 1 mM EDTA), physiological solution (0.9% NaCl) or PBS-buffer. Because PBS-buffer gave the best results, this solution was used as solvent (data not shown).

**Figure 3:**
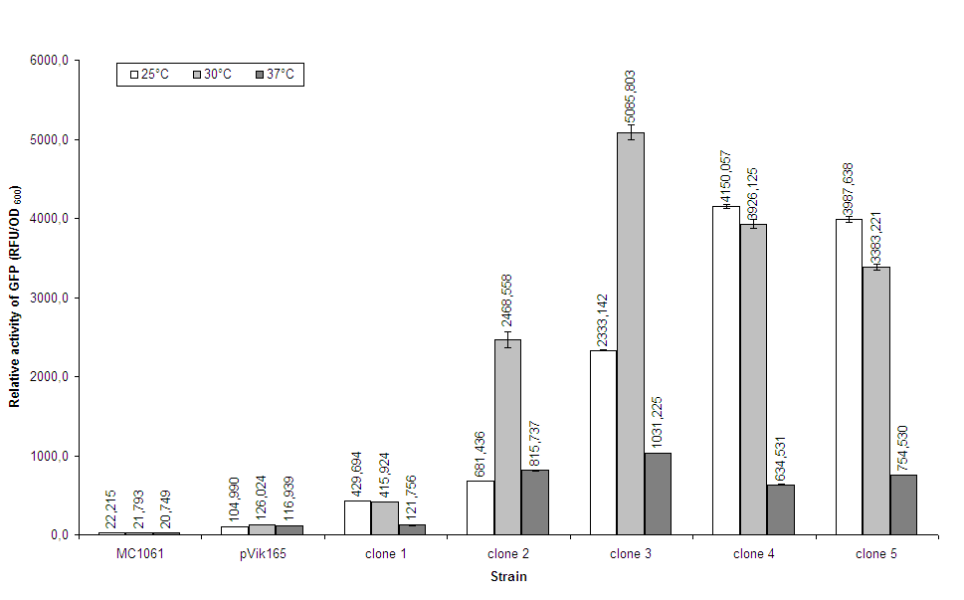
Relative GFP activity for *E. coli *MA8 and synthetic promoter clones for different incubation temperatures.

In a next step the effect of incubation temperature was investigated. Therefore, MA8, MA8 with pVIK165 and 5 clones were grow in 100 ml LB at 25°C, 30°C and 37°C in duplicate. From these cultures 40 ml was harvested and the pellet was washed with 40 ml PBS-buffer and resolved in 4 ml PBS-buffer. Black 96-well microtiter-plates were filled with 100 μl mixture (in fourfold) and readings were carried out at 489 nm excitation wavelength and 511 nm emission wavelength with auto cut off on a Spectramax Gemini XS. Results are given in figure 3.

Figure 3 nicely illustrates that the fluorescence of GFP strongly decreases when *E. coli *is grown at 37°C. *E. coli *expressing GFP shows stronger fluorescence when grown at 25°C or 30°C. This confirms that the formation of the GFP chromophore is temperature sensitive, although the fluorescent GFP is highly thermostable. Thus, we decided to grow *E. coli *at 30°C for GFP activity measurements.

### Activities of the synthetic promoters in *E. coli*

**Figure 4:**
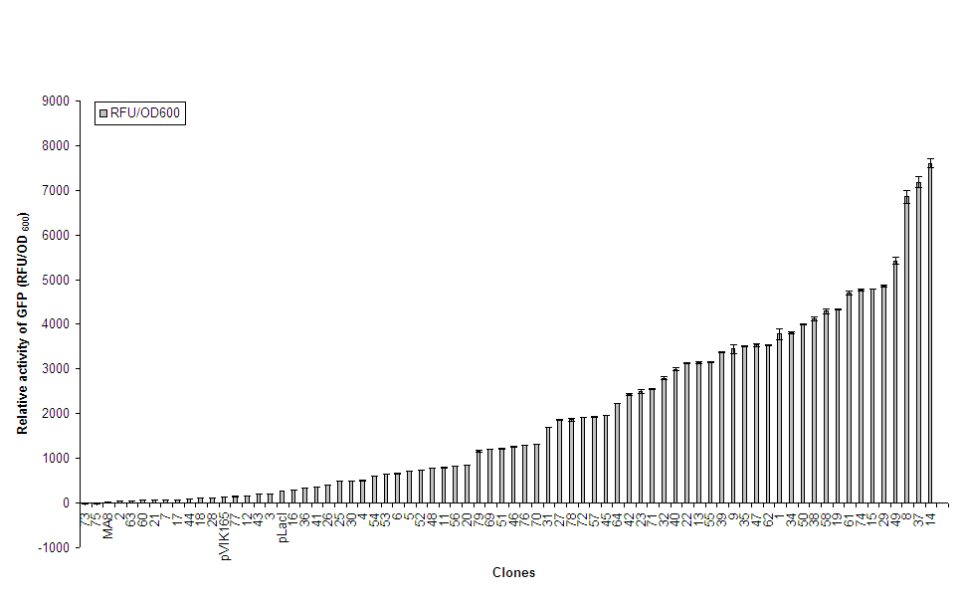
Library of artificial promoters for *E. coli*. Promoter activity (units/OD_600 _ml) was assayed for a reporter gene encoding GFP, transcribed from the synthetic promoter clones.

From the LB/kanamycin agarplates 80 clones were picked up and screened for GFP activity, but only 71 clones seemed to be positive. The green fluorescent protein activity of liquid cultures of these clones was measured; the clones cover a wide range of promoter activities from 21.79 RFU/OD_600_. ml (slightly above the activity found in *E. coli *MA8 without mGFP) up to 7606.83 RFU/OD_600_. ml (see figure 4).

**Figure 5:**
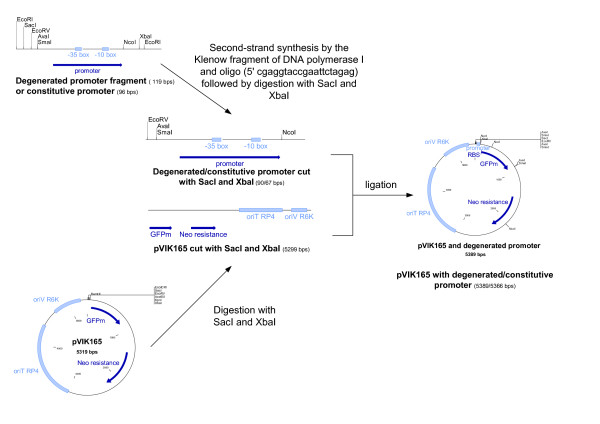
Strategy used for cloning degenerated oligonucleotide promoter fragments/the constitutive promoter *pLacI *into the vector pVIK165.

The promoter library covers 3 to 4 logs of promoter activity in small steps of activity change. The fluorescence from the mGFP gene without a promoter and from *E. coli *MA8, that contains no mGFP gene, was also determined. Furthermore, the activity of the artificial promoters was compared to the activity of the *pLacI *promoter, the constitutive *E. coli *promoter of *LacI*. Therefore, an oligo was synthesized containing the *pLacI *sequence flanked with the same MCS as the degenerated promoter oligonucleotides. In the next step, the *pLacI *oligo was converted into double-stranded DNA and cloned into the pVIK165 vector as described elsewhere (see figure 5).

Some of the promoters which resulted from this approach, turned out to be very strong (more than 27,5-fold the *pLacI*-promoter), others quite weak (almost 7-fold lower than the *pLacI*-promoter). Moreover, circa 4 out of 5 of these artificial promoters have strengths higher than the constitutive *E. coli pLacI*-promoter.

These results confirm the findings from[12,13]. They constructed a library of synthetic promoters for *Lactococcus lactis *that also covered 3 to 4 logs of promoter activity. Thus, their proposed strategy to construct an artificial promoter library of different strengths is indeed effective and here confirmed.

### Promoter sequence analysis

Being able to rationally designing a promoter would be extremely profitable in the context of a model-based metabolic engineering. The present contribution therefore attempts to link the promoter sequence to its strength. To this end, several strategies have been applied.

**Figure 6:**
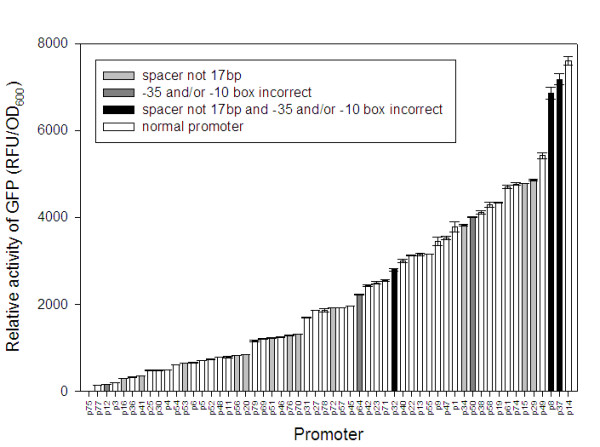
mGFP activities of the synthetic promoters in *E. coli*. The colors of the bars indicate which promoter clones contained errors in either the -35 or the -10 consensus sequence or in the length of the spacer between these sequences.

Firstly, the promoters were subdivided into 4 classes, according to their sequence. Considering the findings of Jensen and Hammer (1998b) and Rud et al. (2006) [13,15] these classes are: 1) promoters affected in their spacer size, 2) promoters affected in their -35 or -10 box, 3) promoters affected both in their spacer size and in their -35 or -10 box, and 4) no apparent anomalies in their sequence. The promoters were ordered according to their strength and coloured according to the class they belong to; the results are depicted in figure 6.

No correlation could be detected between promoter strength and anomalies in the consensus sequence nor the spacer length. This finding for *E. coli *promoters confirms the results of Jensen and Hammer (1998b) [13].

E.g., Promoters 8, 16, and 37 are typical illustration of promoters that do not comply to the postulates of Jensen and Hammer (1998b) and Rud et al. (2006) [13,15]. Though, promoters 8 and 37 have a mutation in the -35 (TTACA) or the -10 box (TATAT), respectively, and both have a spacer with length 17 these promoters are strong. Promoter 16, which belongs to class 4, is on the contrary weak.

The present results thus conclusively show that postulates for other prokaryotes [13,15] linking promoter strength-entirely- to anomalies in the -10 box, -35 box, and the length of the spacer are not generally valid. This might even seem logical as in general overproduction is not in the cell's interest, e.g. the presence of the regulatory mechanisms (feed back, feed forward) in cells preventing overproduction. A rough classification in the proposed classes thus seems not sufficient as a means to rationally design a library of promoters covering a wide range of promoter strengths.

**Figure 7:**
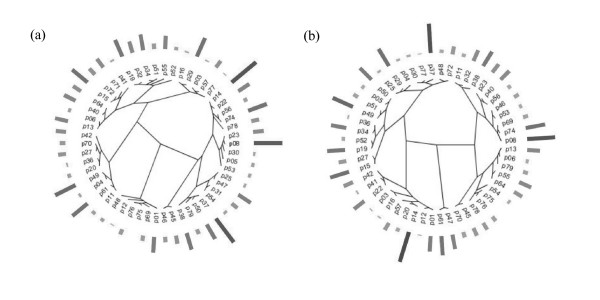
Sequence analyses of the synthetic promoter clones. Each entry represents a promoter. The length of the rectangle equals the promoter strength. (a) total promoter sequence. (b) spacer sequence.

In a second attempt, a more rigorous method was followed to link the promoter sequence to their strength. The sequences of the individual promoters and spacers, respectively, were aligned and compared with the overall promoter strength (figure 7). Each entry represents a promoter. The length of the rectangle equals the promoter strength.

Again no pattern could be detected. The strengths appear to be randomly distributed in the phylogenetic tree. Thus, no clear relationship could be detected between the strength of the promoter and the degree of alignment.

**Figure 8:**
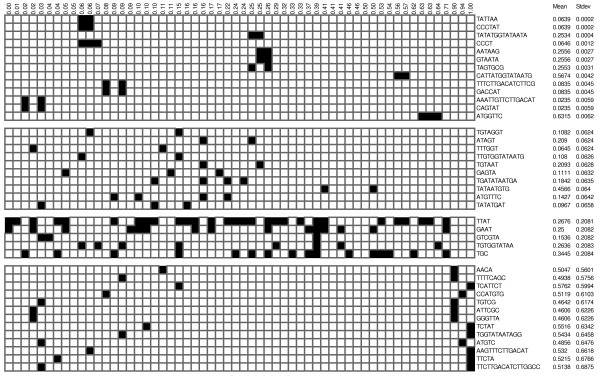
Sequences occurring in the different promoters. The promoters are ordered from low to high; their strength is given at the top. A black square is for substrings that occur in the corresponding promoter. The mean strength (and standard deviation) of each substring is calculated from the different promoters it occurs in.

One could also look at which substrings typically occur in highly expressing promoters and which in low expressing promoters. All possible substrings that occur in more than one promoter and that contain 3 or more nucleotides, were therefore generated. 1216 unique substrings were found. Both the mean of the strengths of the promoters containing such a substring as well as the standard deviation were calculated. If a substring occurs only in promoters that are proximal in strength, the corresponding standard deviation will be low. Thus the substrings were sorted according to the standard deviation of their mean strength. Figure 8 shows some of these substrings.

It was not possible to identify a substring that could be attributed to a certain region of promoter strength. The first block in figure 8 shows the substrings with the lowest standard deviations. Those substrings could be interesting as a low standard deviation means that they occur in promoters that are near each other but unfortunately they do not occur in more than 2 or 3 promoters and thus not really represent a substring typical for a certain promoter strength.

The second and third block of figure 8 shows the results of substrings that occur in more promoters, but spread over the whole promoter strength range causing the standard deviation to be higher. The fourth block shows the substrings with the highest standard deviations. The substrings do not occur often, but very disparately in the promoter strength space.

It seems thus that the strength of a promoter in *E. coli *is not determined by the presence or the absence of certain substrings. However, a sample set of 58 promoters is not enough to conclude that consensus sequences typical for strong or weak promoters do not exist, but if such sequences exist, they are certainly not unique as a random sample of 58 promoters is not enough to find some of them.

In the paper by Jensen et al (2006) [29], a method is given for finding nucleotide positions that have an influence on the strength of promoters. They apply it to a promoter set containing only transitions, no transversions but the method can also be used in the more general case of random mutations. The promoters are divided into two classes: strong and weak, with each class containing n_s _and n_w _promoters, respectively. For each position in the promoter, a count is made of which nucleotides are present in the strong class and which in the weak class (q_s _and q_w_). If a certain nucleotide is found q times across all the promoters at a certain position, it is expected to occur q n_s_/(n_s _+ n_w_) in the set of strong promoters and q n_w_/(n_s _+ n_w_) in the set of weak promoters if there is no correlation between the nucleotide/position combination and the strength of a promoter.

The probability of finding the nucleotide under consideration follows a binomial distribution with chance p and population size q. The probability that a nucleotide at that certain position occurs more than x times in a certain class i is:

Pr⁡(qi≥x)=∑j−xq(qj)pkj(1−pk)q−j
 MathType@MTEF@5@5@+=feaafiart1ev1aaatCvAUfKttLearuWrP9MDH5MBPbIqV92AaeXatLxBI9gBaebbnrfifHhDYfgasaacH8akY=wiFfYdH8Gipec8Eeeu0xXdbba9frFj0=OqFfea0dXdd9vqai=hGuQ8kuc9pgc9s8qqaq=dirpe0xb9q8qiLsFr0=vr0=vr0dc8meaabaqaciaacaGaaeqabaqabeGadaaakeaacyGGqbaucqGGYbGCcqGGOaakcqWGXbqCdaWgaaWcbaGaemyAaKgabeaakiabgwMiZkabdIha4jabcMcaPiabg2da9maaqahabaWaaeWaaeaafaqabeGabaaabaGaemyCaehabaGaemOAaOgaaaGaayjkaiaawMcaaiabdchaWnaaDaaaleaacqWGRbWAaeaacqWGQbGAaaGccqGGOaakcqaIXaqmcqGHsislcqWGWbaCdaWgaaWcbaGaem4AaSgabeaakiabcMcaPmaaCaaaleqabaGaemyCaeNaeyOeI0IaemOAaOgaaaqaaiabdQgaQjabgkHiTiabdIha4bqaaiabdghaXbqdcqGHris5aaaa@52C3@

in which p_k _is equal to the probability that that the nucleotide under consideration is found in the class i given there is no correlation between nucleotides and promoter strength:

pk=nkns+nw
 MathType@MTEF@5@5@+=feaafiart1ev1aaatCvAUfKttLearuWrP9MDH5MBPbIqV92AaeXatLxBI9gBaebbnrfifHhDYfgasaacH8akY=wiFfYdH8Gipec8Eeeu0xXdbba9frFj0=OqFfea0dXdd9vqai=hGuQ8kuc9pgc9s8qqaq=dirpe0xb9q8qiLsFr0=vr0=vr0dc8meaabaqaciaacaGaaeqabaqabeGadaaakeaacqWGWbaCdaWgaaWcbaGaem4AaSgabeaakiabg2da9maaleaaleaadaqfqaqabWqaaiabdUgaRbqab4qaaiabd6gaUbaaaSqaamaavababeadbaGaem4CamhabeGdbaGaemOBa4gaaSGaey4kaSYaaubeaeqameaacqWG3bWDaeqaoeaacqWGUbGBaaaaaaaa@3B10@

where n_k _is the number of promoters in the class under consideration.

**Figure 9:**
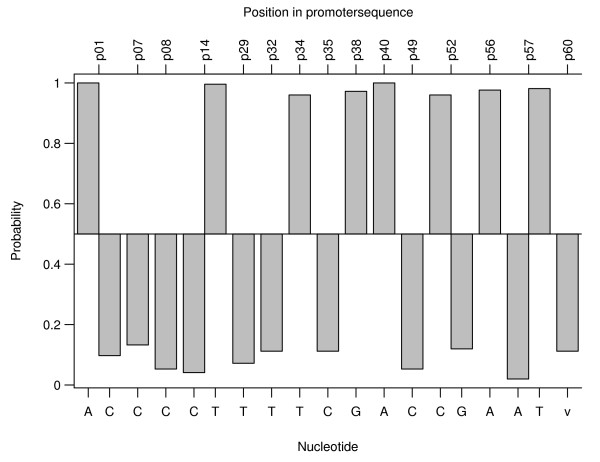
Probability (y-axis) that a certain nucleotide (bottom x-axis) at a certain position in the promoter (top x-axis) occurs more than it actually occurs in the promoters classified as strong, assuming it follows a binomial distribution (the data for the weak class are similar). 16 promoters were classified as strong and 42 as weak. Only position/nucleotide combinations with more than 5 nucleotides and with a P-value less than 0.05 were retained. Thus a position/nucleotide combination with a high probability means that this nucleotide does not occur often in promoters classified as strong. The v nucleotide, v standing for void, at position 60 means no nucleotide present at that position.

When the actual occurrence of a nucleotide at a certain position in a certain class, is less than the expected occurrence, the probability calculated by equation 1 will be greater than 0.5. It will be less than 0.5 when a nucleotide is overrepresented. In figure 9, only position/nucleotide pairs that have a high P-value are shown. The P-value is defined as:

P−value={Pr⁡(qi≥x)1−Pr⁡(qi≥x)if Pr⁡(qi≥x)>0.5if Pr⁡(qi≥x)≤0.5
 MathType@MTEF@5@5@+=feaafiart1ev1aaatCvAUfKttLearuWrP9MDH5MBPbIqV92AaeXatLxBI9gBaebbnrfifHhDYfgasaacH8akY=wiFfYdH8Gipec8Eeeu0xXdbba9frFj0=OqFfea0dXdd9vqai=hGuQ8kuc9pgc9s8qqaq=dirpe0xb9q8qiLsFr0=vr0=vr0dc8meaabaqaciaacaGaaeqabaqabeGadaaakeaafaqabeqacaaabaGaemiuaaLaeyOeI0IaemODayNaemyyaeMaemiBaWMaemyDauNaemyzauMaeyypa0ZaaiqaaeaafaqabeGabaaabaGagiiuaaLaeiOCaiNaeiikaGIaemyCae3aaSbaaSqaaiabdMgaPbqabaGccqGHLjYScqWG4baEcqGGPaqkaeaacqaIXaqmcqGHsislcyGGqbaucqGGYbGCcqGGOaakcqWGXbqCdaWgaaWcbaGaemyAaKgabeaakiabgwMiZkabdIha4jabcMcaPaaaaiaawUhaaaqaauaabeqaceaaaeaaieGacqWFPbqAcqWFMbGzcqqGGaaicyGGqbaucqGGYbGCcqGGOaakcqWGXbqCdaWgaaWcbaGaemyAaKgabeaakiabgwMiZkabdIha4jabcMcaPiabg6da+iabicdaWiabc6caUiabiwda1aqaaiab=LgaPjab=zgaMjabbccaGiGbccfaqjabckhaYjabcIcaOiabdghaXnaaBaaaleaacqWGPbqAaeqaaOGaeyyzImRaemiEaGNaeiykaKIaeyizImQaeGimaaJaeiOla4IaeGynaudaaaaaaaa@7313@

The higher the P-value, the more likely the corresponding position/nucleotide combination influences the promoter strength.

Whereas the former techniques did not show any correlation between promoter sequence and promoter strength, this technique clearly shows that such a correlation exists: some positions could be identified as having a high influence on promoter strength. Interesting to note is that promoters classified as strong, have a tendency to be shorter than those classified as weak (see figure 9).

**Figure 10:**
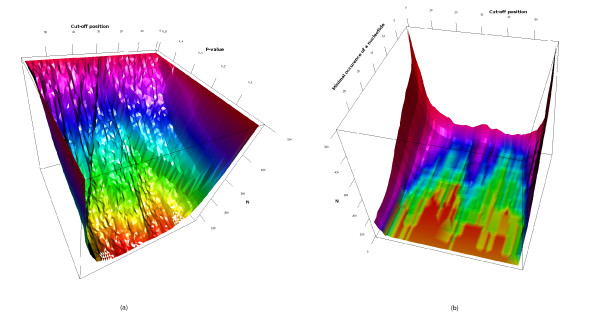
(a) Number of significant position/nucleotide combinations found in the strong and the weak class (N) in function of the cut-off position and maximal P-value. (b) Number of significant position/nucleotide combinations found in the strong and the weak class (N) in function of the cut-off position and the minimal number of times a nucleotide at a certain position should occur before that position/nucleotide combination is considered. The maximal P-value was 0.05.

But the technique has some shortcomings in addition to the drawbacks mentioned by Jensen et al. (2006) [29]: 1) Promoters are classified in (two) classes. One would prefer a model that predicts the promoter strength quantitatively and not only qualitatively. 2) The artificial division into two classes: were should the cut-off value be chosen? One big class and one small class? Or both classes with the same size? Figures 10a and 10b show the sensitivity of the number of position/nucleotide combinations retained as having influence on the promoter strength on the cut-off value (place were the promoter strength region is splitted). In figure 10a it can be seen that the number of position/nucleotide combinations that are retained as having influence on the promoter strength is variable when the cut-off value changes for a constant maximal P-value. 3) Some position/nucleotide combinations have more influence on the classification than others. But when is a site considered to have influence and when not? Which maximal P-value should be chosen? Both figure 10a and 10b show that the maximal P-value has a significant influence on the position/nucleotide combinations retained. 4) The influence of the number of nucleotides that should be counted before any influence of a nucleotide at a certain position can be assessed: when only two promoters have a certain nucleotide at a certain position and both of them are in the same class, that nucleotide/position combination will appear to have a strong influence on the promoter strength. However, a nucleotide that occurs only two times is not really representative and should thus not be considered.

More generally, what is the minimal occurrence required for a nucleotide (over both classes) at a certain position before considering to test it for influence on promoter strength? Figure 10b shows that the number of relevant position/nucleotide combinations drops significantly when the minimal occurrence requested increases. Also, when the requested minimal occurrence is higher, the influence of the cut-off position is stronger: more significant position/nucleotide combinations are found when the cut-off position is in the middle or at one of the ends of the promoter strength region.

Nevertheless, this technique can aid in designing new promoters, as was shown by Jensen *et al. *(2006) [29]. But it will never be very quantitative, due to the classification of the promoters. Partial Least Squares (PLS) models do not have the above mentioned limitations.

Therefore, to link the promoter sequence to its strength in a quantitative way, PLS regression has been performed. This generalization of multiple linear regression is able to analyze data with strongly collinear and numerous independent variables as is the case for the promoter library under study. Partial least squares regression is a statistical method that links a matrix of independent variables, X, with a matrix of dependent variables, Y, i.e. the nucleotide sequence and the promoter strength, respectively. Therefore, the multivariate spaces of X and Y are transformed to new matrices of lower dimensionality that are correlated to each other. This reduction of dimensionality is accomplished by principal component analysis like decompositions that are slightly tilted to achieve maximum correlation between the latent variables of X and Y [30].

The nucleotide sequences were therefore encoded in the matrix X, as shown in Table 1. Each entry in a column represents the absence (0) or presence (1) of a certain nucleotide at a certain position for a certain promoter [31]. Prior to further evaluations, the columns containing only zeros or only ones were eliminated.

**Table 1:** Construction of the matrix X. Each nucleotide at each position occupies a column.

Position	1	1	1	1	2	2	2	2
Nucleotide	A	C	G	T	A	C	G	T

Promoter 1	1	0	0	0	0	1	0	0
Promoter 2	0	0	0	1	1	0	0	0
Promoter 3	0	0	1	0	1	0	0	0
Promoter 4	0	1	0	0	0	1	0	0

The data set was randomly divided into two parts: the training set, containing 42 of the 49 promoters, and the test set, containing 7 of the 49 promoters. The PLS model was then built using the training set. First, to avoid overfitting – as this would result in a model not able to generalize to new data-cross-validation was applied to determine the appropriate number of latent variables. In cross-validation the data X_training_, and Y_training_, are split into blocks and a one latent variable model is built from (k-1) blocks of data. Based on this model, the excluded block is used for testing and an individual predictive relative error sum of squares, PRESS, is calculated. This procedure is repeated excluding each block once, and the total PRESS is calculated for the model with one latent variable. This procedure is then repeated for 2, 3, ... min (m, n) latent variables, with n and m the sample size and the number of variables, respectively, and a series of PRESS values are obtained [32]. Wold's R criterion, given as R = PRESS(i+1)/PRESS(i) ≤1, was applied to determine the number of latent variables to be used in the final model. An additional latent variable is retained only when R is smaller than one [33]. Using this procedure, 5 latent variables were retained in the PLS model.

**Figure 11:**
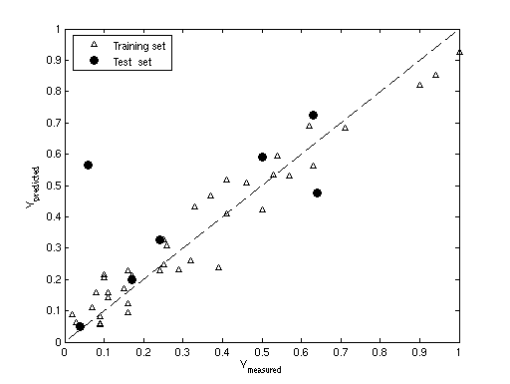
The predicted promoter strength (y predicted) versus the observed promoter strength (y measured) of the training set and the test set.

Sequentially, the model's predictive ability was assessed. To this end the promoter strengths of the promoters in the test set were predicted by the fully trained PLS model. The predicted strength versus the observed strength is depicted in Figure 11.

Thus, a PLS model was built that correlates the promoter strength to its sequence. The validity of the approach is shown by the ability to make external predictions: the strength of 6 out of 7 promoters of the test set is reasonably well predicted.

## Conclusion

An artificial constitutive promoter library was synthesized. The mGFP activity of liquid cultures of *E. coli *MA8 cells in which those promoters were transformed, range from 22 RFU/OD600 to 7600 RFU/OD600. 57 promoters were sequenced and used for promoter analysis.

No correlation could be detected between promoter strength and anomalies in the spacer length of the -10 and/or -35 box. This in contrast to the findings of Jensen and Hammer (1998b) and Rud et al. (2006) [13,15] for other prokaryotes, but agrees with the results for *E. coli *found by Jensen and Hammer (1998b) [13].

No clear relationship could be established between promoter strength and degree of alignment and in addition no typical substrings could be detected for strong or weak promoters.

The method of Jensen et al. (2006) [29] has been applied to find position/nucleotide combinations that are significantly influencing the promoter strength. Positions could be detected were the choice of nucleotides is likely to influence promoter strength. But the technique appears to be sensitive to the maximal P-value and on how the promoters are splitted into two classes. Furthermore predictions are of limited use, as they only give qualitative information: "strong" or "weak".

A PLS model was built and validated that reasonably well correlated the promoter strength to its sequence. Such a model can be an extremely useful tool to rationally design a suitable promoter in order to fine tune gene expression in the framework of model-based metabolic engineering.

## Methods

### Bacterial strain and plasmids

*Escherichia coli *MA8 [supE, thi, del(lac-proAB), λ-(pir116, on F')] was obtained from the Netherlands Culture Collection of Bacteria (NNCB). MA8 was used to study promoter activities in *Escherichia coli *as well as for cloning purposes. The vector pVIK165 was obtained from the Laboratory of Molecular Biotechnology Plasmid Collection (LMBP). pVIK165 is a vector for *Escherichia coli *conferring kanamycin resistance to the host cell. The vector pVIK165 is a so called suicide vector, carrying a "suicide γ-*ori*" replicon. The activity of this origin of replication is restricted to those *E. coli *strains (e.g. *E. coli *MA8) that can provide *in trans *the π-protein encoded by the *pir *gene. The vector carries no promoter followed by a prokaryotic synthetic ribosome binding site (RBS, 5'-ggagga) and a variant of the green fluorescent protein (mGFP) gene (containing a threonine residue at amino acid position 65 instead of wild type serine residue).

### Culture conditions

The culture medium Luria Broth (LB) consisted of 1% tryptone peptone (Difco^®^), 0.5% yeast extract (Difco^®^), and 0.5% sodium chloride (Esco^®^). The pH of the medium was 6.7. A seed culture in 5 ml of the medium in a 20 ml test tube was grown overnight and 2 ml was transferred to 100 ml medium in a 0.5 l Erlenmeyer flask. Incubation was performed at 37°C and at 200 rpm for 12 hours.

### Enzymes and oligonucleotides

Restriction enzymes, Klenow DNA polymerase, and T4 DNA ligase were obtained from and used as recommended by Roche^® ^and Fermentas^®^, respectively.

Oligonucleotides were obtained from Sigma Genosys^®^.

### Second-DNA-strand synthesis and cloning of synthetic DNA fragments into the vector pVIK165

The single-stranded degenerated promoter oligonucleotides were converted to double-stranded DNA using a 20 bp oligonucleotide (5'-cgaggtaccgaattctagag) complementary to the 3' end of the degenerated promoter oligonucleotide as primer for the second-strand synthesis by the Klenow fragment of DNA polymerase I.

The cloning strategy used, is explained in figure 5. The mixture of degenerated promoter oligonucleotides and pVIK165 were digested with restriction enzymes SacI and XbaI. As a result the pVIK165 was cut before the RBS and the degenerated promoter fragments were ligated to the compatible vector fragments. The ligation mixtures were then transformed into Ca^2+^-competent MA8 cells by using a modified transformation procedure of [34]. The transformation mixtures were plated on LB agar plates containing (per liter) 0.025 g kanamycin.

### Cloning the constitutive p*LacI *promoter into the vector pVIK165

The single-stranded *LacI *promoter oligonucleotide (5'-ggcgcaaaacctttcgcggtatggcatgatagcg) flanked with the same MCS of the degenerated promoters were converted to double-stranded DNA using a 20 bp oligonucleotide (5'-cgaggtaccgaattctagag) complementary to the 3' end of the MCS as primer for the second-strand synthesis by the Klenow fragment of DNA polymerase I.

The cloning strategy used, is revealed in figure 5. The p*LacI *oligonucleotide and pVIK165 vector were digested with restriction enzymes SacI and XbaI. As a result the pVIK165 was cut before the RBS and the *LacI *promoter was ligated to the compatible vector fragment. The ligation mixture was then transformed into Ca^2+^-competent MA8 cells using a modified transformation procedure of Hanahan (1991) [34]. The transformation mixtures were plated on LB agar plates containing (per liter) 0.025 g kanamycin.

### Green fluorescent protein assay

An assay for the mutated green fluorescent protein was developed based on the GFP assay described by Clontech Laboratories (1999) and Gonzales (2005) [27,28]. Cultures carrying the plasmid derivates of pVIK165 were grown in duplicate in LB supplemented with kanamycin. After determination of the optical density at 600 nm (OD_600_) with an UVIKOM 922, 40 ml culture was harvested and the pellet was washed with 40 ml PBS-buffer (20 mM phosphate buffer pH 7.4, 150 mM NaCl) and resolved in 4 ml PBS-buffer. Black 96-well microtiter-plates were filled with 100 μl mixture (in fourfold) and readings were carried out at 489 nm excitation wavelength and 511 nm emission wavelength with auto cut off on a Spectramax Gemini XS.

### Promoter sequence analysis

Plasmids were extracted using a Nucleospin^® ^Plasmid Quick Pure kit (Macherey-Nagel) and sent to the Genetic Service Facilities for sequencing using a 21 bp oligonucleotide (5'-taaccttcgggcatggcactc) complementary to the mGFP gene. A multiple sequence alignment was done using the software ClustalW. The software package TreeIllustrator was applied to visualize a radial tree and to link the respectively promoter activity to each entry [35]. Substring analysis and influence of nucleotide/position combinations on promoter strength as described by Jensen et al. (2006) [29], were done in Perl. Plots were generated in R [36] using the rgl package [37]. Partial Least Squares (PLS) regression was done in the software package R [36].

## Authors' contributions

MDM carried out the molecular genetic studies and participated in the sequence alignment. GJL carried out the probabilistic approach to relate the promoter sequence to its strength. JM applied PLS regression to construct a model. MDM, GL and JM drafted the manuscript. EJV and WKS revised the manuscript critically. All authors read and approved the final manuscript.
